# Bar-Related Infections After Minimally Invasive Repair of Pectus Excavatum: A 25-Year Single-Center Retrospective Cohort Study

**DOI:** 10.1055/a-2888-9665

**Published:** 2026-06-15

**Authors:** Hendrik van Braak, Erika P. M. van Elzakker, Sjoerd A. de Beer, Matthijs W. N. Oomen, Sander Zwaveling, Lodewijk W. E. van Heurn, Justin R. de Jong

**Affiliations:** 1 Department of Pediatric Surgery 332563 Emma Kinderziekenhuis Amsterdam UMCAmsterdamNetherlands; 2 Department of Medical Microbiology and Infection Prevention 522567 Amsterdam University Medical CentresAmsterdamNetherlands

**Keywords:** Nuss procedure, postoperative infection, cutibacterium acnes, bar dislocation, infection management

## Abstract

**Introduction:**

This study aims to evaluate the incidence and characteristics of Nuss bar infections, identify factors associated with infection, and present our institutional management considerations.

**Methods:**

We conducted a single-center retrospective cohort study of patients undergoing the Nuss procedure between 1999 and 2024 at Amsterdam Pectus Center. All patients had a minimum follow-up of 6 months. Primary outcomes included incidence and management of infections. Secondary outcomes included infection characteristics (early- [<30 days] vs. late-onset [>30 days], superficial vs. deep), diagnostics, microbiology, factors associated with infection (sex, age, type of anesthesia, number of bars, bar dislocation, stabilizer position, use of sutures), impact on bar removal, incidence of allergies and terra firma-forme dermatosis.

**Results:**

Of 695 patients, 4.0% ( *n* = 28/695) developed Nuss bar infections, at a median of 2.0 (0.0–13.5) months postoperative, presenting with erythema, pain, and exudate. Over time, infections shifted from early- to late-onset ( *p* = 0.03). Deep infections (71.4%, *n* = 20/28) more often required surgery ( *p* = 0.007); superficial infections were managed with a wide range of antibiotics. Bar dislocations (5.6%, *n* = 39/695) and stabilizer plate loosening (1.0%, *n* = 7/695) required reoperation and were associated with infections ( *p* = 0.001). Operative time for bar removal was longer in infected patients (52.0 [32.0–68.0] vs. 35.0 [26.0–49.0] minutes, *p* = 0.03). One case of cobalt allergy was identified. Terra firma-forme dermatosis (0.4%, *n* = 3/695) was treated with alcohol wipes.

**Conclusion:**

Bar dislocation and stabilizer plate loosening are associated with Nuss bar infections. We present our institutional management considerations based on retrospective data and experience, requiring prospective validation. Accurate diagnostics, tailored antibiotic therapy, and consideration of alternative diagnoses remain essential.

## Introduction

Pectus excavatum (PE) is a chest wall anomaly for which minimally invasive repair (MIRPE), first described by Nuss et al, 1 is the most common treatment. 2 Despite favorable outcomes, postoperative infections remain a concern, with an incidence of 1.5 to 9.4%. 3 4 5 6 7 8 9 10 11 12 13 14 15 Affected patients often require extended antibiotic therapy and occasionally, additional surgical interventions, although the Nuss bar is typically retained. 4 13 16 The most comprehensive study on postoperative infections following MIRPE, including a protocol, was published in 2018. 4

Since then, the surgical technique of the Nuss procedure has advanced significantly. In particular, the introduction of intercostal nerve cryoablation and changes in fixation techniques and implant design may have influenced the presentation and microbiology of postoperative infections, potentially affecting the applicability of existing diagnostic and management strategies derived from earlier cohorts. 17 In our clinical practice, we observed an increase in low-grade, late-onset postoperative infections alongside these advancements. These observations prompted us to investigate whether the characteristics and microbiology of Nuss bar infections have changed over time, particularly with a shift toward late-onset, low-grade infections with Cutibacterium acnes (CA), formerly Propionibacterium acnes, and whether this has implications for current diagnostic and management strategies.

We therefore evaluated their incidence, characteristics, and temporal trends in a 25-year cohort, and describe our institutional management considerations, including differentiation from alternative diagnoses, as these may clinically resemble infection but require different management.

## Methods

### Study Population and Design

We performed a retrospective cohort study of patients undergoing MIRPE in the Amsterdam Pectus Center, Amsterdam University Medical Center, between March 1999 and April 2024. The Medical Ethics Review Committee of the Amsterdam Medical Center waived ethical approval. Informed consent was obtained from all patients (or their parents).

### Data Extraction

Data were retrospectively obtained from patient records. Infections were defined as cellulitis that required antibiotics, superficial infection with active drainage, or deep infections involving hardware, regardless of onset. 4 An infection was considered deep if ultrasound confirmed bar involvement, the bar or stabilizer plate became exposed, or imaging or surgery revealed direct connection between the infection and the bar (fistula/abscess). Diagnostic criteria (clinical, imaging, and microbiological) were not fully standardized over the study period.

Exudate was defined as purulent drainage. A 30-day threshold differentiated between early-onset and late-onset infections. Operative time for Nuss bar removal was defined as the time between incision and wound closure. Rotation, lateral shift, flipping, or intrathoracic displacement are all defined as Nuss bar dislocation. Stabilizer plate loosening is defined as fixation loss, allowing plate migration along the bar. Nuss bar allergy had to be confirmed by a dermatologist through standardized patch testing.

Loss to follow-up was minimal due to routine follow-up until bar removal, and no missing data were present for the variables included in the analyses.

### Treatment Algorithm

**Fig. 1:**
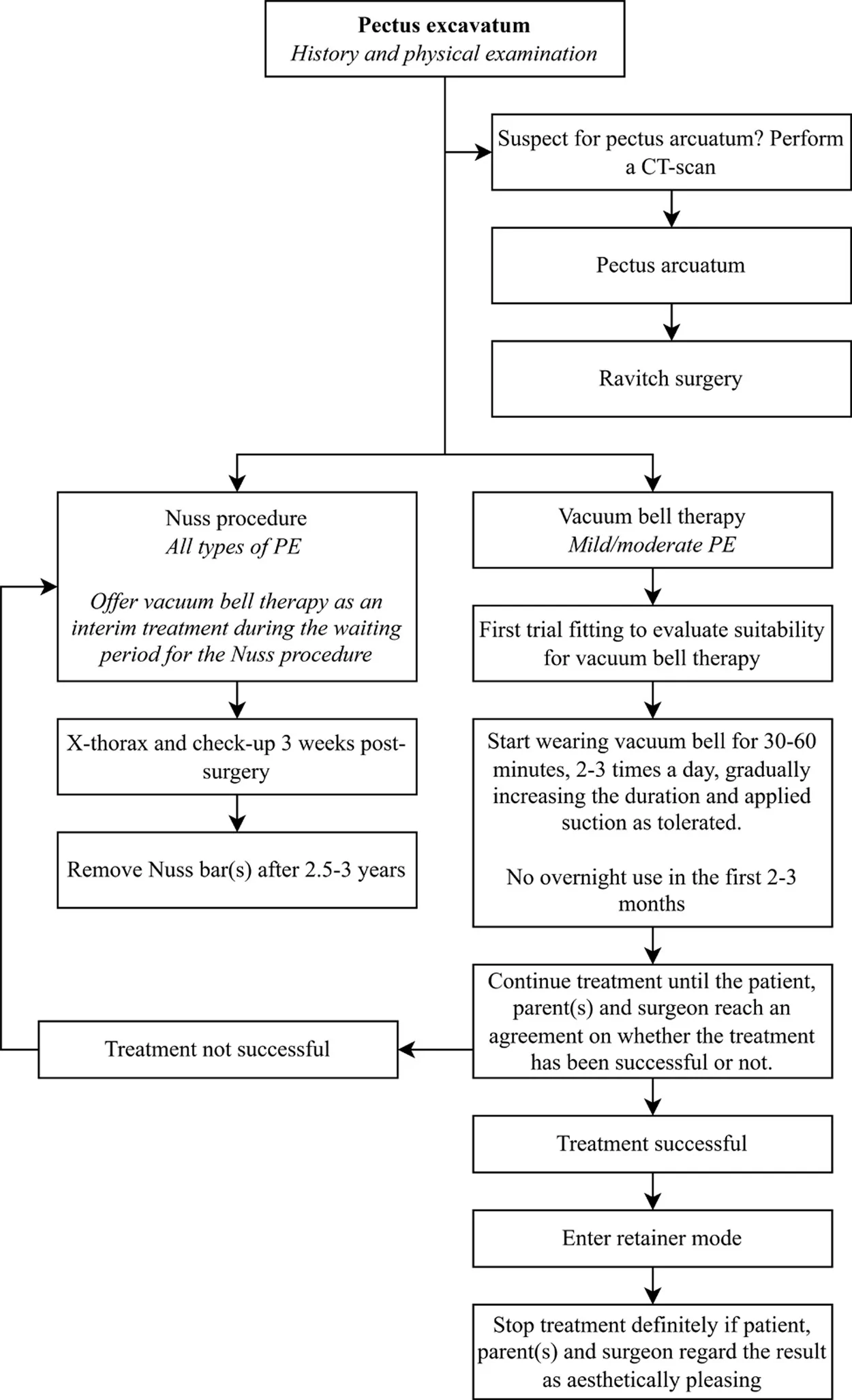
Management algorithm for pectus excavatum. PE, Pectus excavatum.

Fig. 1 shows our treatment algorithm. Routine allergy testing is not part of our protocol. Until 2019, patients undergoing the Nuss procedure received continuous epidural analgesia combined with patient-controlled analgesia. Since then, the procedure has been performed using intercostal nerve cryoablation combined with patient-controlled analgesia for the first 24 hours after surgery. 17

All patients receive intravenous cefazolin for 24 hours, starting 30 minutes before the first incision (adolescents: 3 × 1,000 mg/day, children: 3 × 100–150 mg/kg, maximum of 6 g/day). We follow the Dutch and international guidelines regarding surgical site infection prevention. 18 19 Scrubbing is done twice with chlorhexidine/alcohol skin antiseptic solution.

We perform MIRPE using a modified version 20 of the original technique. 1 Typically, one Nuss bar is used, although sometimes two bars are required. A single stabilizer plate on the left side of the bar, as medially as possible on the chest wall, secures the bar(s). In the early years, the bar (Biomet) was sutured to the stabilizer plate on one side, and secured with a thoracoscopic placed pericostal, absorbable suture on the other side. We later adopted bars (MedXpert) that featured a stabilizer plate with screws, making sutures redundant. We recommend leaving the bar in situ for 3 years.

To account for temporal changes in surgical practice, key variables such as year of surgery, fixation technique (left or right stabilizer), and analgesia were analyzed individually rather than grouped by time period. Year of surgery was used as a proxy for implant type, as the transition from Biomet to MedXpert implants occurred gradually over time and implant type was not always documented in operative reports. Follow-up and infection surveillance remained consistent over time.

### Outcomes

Primary outcomes were incidence and management of Nuss bar infections. Secondary, the early- and late onset infections were compared based on annual incidence, C-reactive protein (CRP), leukocyte count, and type of bacteria. Also, the superficial and deep infections were compared based on the need for surgical intervention, hospital admission, wound drainage, type of cultured bacteria, timing of infection, annual incidence, and type and duration of antibiotic treatment. Other secondary outcomes were infection symptoms, used diagnostics, type of cultured bacteria, analysis of factors associated with Nuss bar infections (sex, age, type of anesthesia, number of Nuss bars, Nuss bar dislocation, and place of stabilizer plates and suture use in relation to infection site), Nuss bar removal time after infection, and incidence of allergies and terra firma-forme dermatosis.

### Statistical Analysis

Descriptive measurements were utilized for baseline characteristics. *p* -Value < 0.05 was considered significant. Data were analyzed using IBM SPSS Statistics 28.0. Distribution of continuous variables was assessed using the Shapiro–Wilk test. Normally distributed variables are reported as mean ± standard deviation (SD), and nonnormally distributed variables are reported as median with interquartile range (IQR).

## Results

All ( *n* = 695) patients who underwent the Nuss procedure were included. Baseline characteristics are displayed in Table 1 . Median age at surgery was 15.0 (14.0–17.0) years; 82.4% of patients were male ( *n* = 573/695). Most patients received one bar (89.8%, *n* = 624/695); 10.1% received two bars ( *n* = 70/695), and 0.1% received three bars ( *n* = 1/695). All patients had a minimum follow-up of 6 months after Nuss bar placement.

**Table 1:** Baseline characteristics

	*n* = 695
Median age at surgery (y) a	15.0 (14.0–17.0)
Sex b	
Male	573 (82.4)
Female	122 (17.6)
Number of bars b	
One	624 (89.8)
Two	70 (10.1)
Three	1 (0.1)
Nuss bar removal b	571 (82.2)
Nuss bar removal after (mo) a	32.0 (29.0–37.0)
Nuss bar infection b	28 (4.0)

a Continues variables expressed as median (IQR); all remaining continuous variables are expressed as mean ± standard deviation.

b Variables displayed as *n* (%).

### Incidence of Nuss Bar Infections

**Fig. 2:**
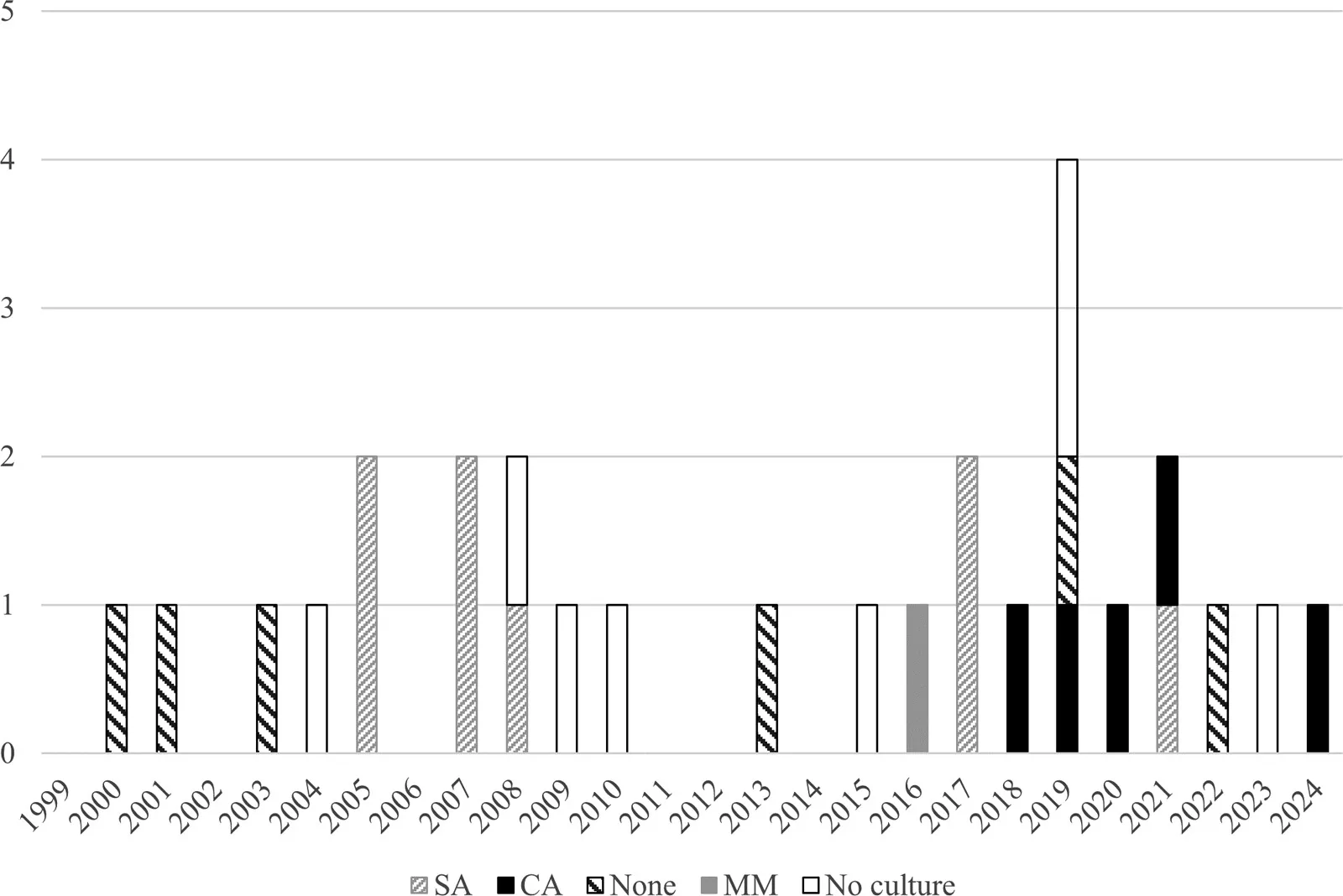
Number of Nuss bar infections per year and cultured bacterial species. CA, Cutibacterium acnes; MM, Morganelli morganii, SA; Staphylococcus aureus.

Of all patients, 4.0% ( *n* = 28/695) developed a Nuss bar infection. Infection-specific details are displayed in Table 2 . Fig. 2 shows infections per year and cultured bacteria. There was no relationship between the year of surgery and the incidence of infections ( *p* = 0.29).

**Table 2:** Infection-specific details

	*n* = 28
*Timing, symptoms and diagnostics*	
Time after Nuss bar placement (mo) a	2.0 (0.0–13.5)
Early onset infection b	10 (35.7)
After surgery for dislocation b	7 (25.0)
Symptoms a	
Erythema	19 (67.9)
Pain	20 (71.4)
Fever	9 (32.1)
Exudate	11 (39.3)
Deep infection b	20 (81.4)
Ultrasound Nuss bar b	15 (53.4)
Thoracic X-ray b	24 (85.7)
Allergy test b	4 (14.3)
Positive (for cobalt)	1 (3.6)
Leukocytes (10 ^9^ /L; *n* = 16) a	10.0 (7.4–13.5)
C-reactive protein (mg/L; *n* = 16) a	17.3 (7.4–64.5)
Eosinophils (10 ^9^ /L; *n* = 10) a	0.13 (0.07–0.34)
Tissue culture taken b	19 (67.9)
Type of bacterium cultured b	
None	5 (17.9)
Staphylococcus aureus	8 (28.6)
Cutibacterium acnes	5 (17.9)
Morganelli morganii	1 (3.6)
*Treatment*	
Patients hospitalized for treatment b	16 (57.1)
Length of stay (d)	9.2 (6.5)
Surgery because of infection b	
One	8 (28.6)
Two	3 (10.7)
Percutaneous drainage b	6 (21.4)
Total duration of antibiotics a	42.0 (14.0–49.0)
Treated with IV antibiotics b	12 (42.9)
Bar removed early b	4 (14.3)

a Continues variables expressed as median (IQR); all remaining continuous variables are expressed as mean ± standard deviation.

b Variables displayed as *n* (%).

### Early versus Late-Onset Infections

The median time between Nuss bar placement and infection was 2.0 (0.0–13.5) months or 8.0 (3.0–41.0) weeks. A significant association was found between the year of surgery and infection timing, with early-onset infections predominating in earlier years and a gradual shift toward late-onset infections in recent years ( *p* = 0.03). Compared with late-onset infections, early-onset infections had higher CRP (mg/L) and leukocyte levels (10 ^9^ L); (CRP: 408.0, 240.0, 223.0, 61.8; leukocytes: 25.8, 23.0, 15.9, 11.9) (CRP: 11.1 [6.2-40.2]; leukocytes: 9.3 [6.7-10.4]).

CA was isolated more frequently in late-onset infections (one vs. four), although this difference was not significant ( *p* = 0.62). Although more early-onset infections occurred in patients with sutures, this difference was not significant ( *p* = 0.24).

### Deep versus Superficial Infections

Twenty patients (71.4%) developed deep infections. None of the superficial infections required surgery, whereas 55.0% ( *n* = 11/20) of deep infections did ( *p* = 0.007). No significant differences were seen between deep and superficial infections in hospital admissions ( *p* = 0.07), wound drainages ( *p* = 0.14), or bacteria cultured ( *p* = 1.0). No correlation was found between deep or superficial infections and the year they occurred ( *p* = 0.75), early and late-onset infections ( *p* = 0.62), or antibiotic treatment duration ( *p* = 0.57).

### Symptoms and Diagnostics

Table 2 shows an overview of symptoms and diagnostic tests. Most patients presented with erythema (67.9%, *n* = 19/28) and pain (71.4%, *n* = 20/28), while fever (32.1%, *n* = 9/28) and exudate (39.3%, *n* = 11/28) were less common. Of patients, 21.4% ( *n* = 6/28) had one symptom, 46.4% ( *n* = 13/28) had two symptoms, and 32.1% ( *n* = 9/28) had three symptoms. Thoracic X-rays and Nuss bar ultrasounds were made in, respectively, 85.7% ( *n* = 24/28) and 53.4% ( *n* = 15/28) of patients. Allergy tests were conducted in 14.3% ( *n* = 4/28), revealing a cobalt allergy in one patient.

### Shift in Bacterial Species

Fig. 2 shows a shift in bacteria over time. CA has replaced Staphylococcus aureus (SA) as the most commonly cultured bacterium since 2018.

### Treatment

Table 2 provides an overview of treatment for Nuss bar infections. Of all patients, 53.6% ( *n* = 15/28) were hospitalized one ( *n* = 15) or two ( *n* = 1) times, and 39.3% ( *n* = 11/28) underwent one ( *n* = 8) or two ( *n* = 3) surgeries. Patients received antibiotics for a median duration of 42.0 (14.0–49.0) days. Antibiotic use was heterogeneous, with flucloxacillin ( *n* = 16) and amoxicillin–clavulanic acid ( *n* = 14) most frequently prescribed, followed by clindamycin ( *n* = 7) and amoxicillin ( *n* = 3). Other antibiotics were used less frequently, including rifampicin ( *n* = 2), gentamicin ( *n* = 1), ceftazidime ( *n* = 1), vancomycin ( *n* = 1), ciprofloxacin ( *n* = 1), cotrimoxazole ( *n* = 1), and pheneticillin ( *n* = 1). Over time, a shift in prescribing patterns was observed, from predominantly amoxicillin–clavulanic acid and flucloxacillin in earlier years to more frequent use of flucloxacillin and clindamycin in recent cases, used sequentially rather than in combination.

In 14.3% ( *n* = 4/28), the Nuss bar was removed early. In two patients this was done after 24 months; in one patient the first bar was removed after 12 months, the second after 22 months. In the last patient, the bar was removed but later replaced.

### Factors Associated with Nuss Bar Infection and Its Impact on Bar Removal Time

Table 3 shows factors associated with infections. Bar dislocation (5.6%, *n* = 39/695) and stabilizer plate loosening (1.0%, *n* = 7/695) caused reoperations in all affected patients and were more prevalent in Nuss bar infections ( *p* = 0.001). Reoperations for bar dislocation, plate loosening, and pain have declined over time ( *p* = 0.02), with an annual rate of 0 to 3.1% in the past 5 years.

**Table 3:** Factors associated with postoperative Nuss bar infections

	No infection ( *n* = 667)	Infection ( *n* = 28)	*p* -Value
Median age at surgery (y) a	15.0 (14.0–17.0)	16.0 (15.0–17.0)	0.06
Sex b			
Male	546 (81.9)	27 (96.4)	0.14
Female	121 (18.1)	1 (3.6)	
Number of bars b			
One	602 (90.3)	22 (78.6)	0.15
Two	64 (9.6)	6 (21.4)	
Three	1 (0.1)	–	
Type of analgesia b			
Cryoanalgesia	98 (14.7)	5 (17.8)	0.71
Epidural analgesia	569 (85.3)	23 (82.1)	
Bar dislocation	21 (3.1)	7 (25.0)	0.001
Year of surgery a	2011 (2005–2018)	2016 (2007–2019)	0.29

a Continues variables expressed as median (IQR); all remaining continuous variables are expressed as mean ± standard deviation.

b Variables displayed as *n* (%).

Stabilizer plates were placed on the left in 60.7% of patients ( *n* = 17/28), on the right in 25.0%, and on both sides in 10.7% ( *n* = 3/28). Infection site and stabilizer plate position were significantly related ( *p* = 0.007).

Operative time for Nuss bar removal was longer in patients with infections 52.0 (32.0–68.0), compared with those without (35.0 [26.0–49.0], *p* = 0.03). Infection rate was not related to year of surgery ( *p* = 0.29), age ( *p* = 0.06), sex ( *p* = 0.14), number of bars ( *p* = 0.15), or type of analgesia ( *p* = 0.71).

### Skin Reactions

Aside from bar infections, three patients were suspected of being allergic. Two experienced allergy-like symptoms, although no allergy was found. One patient's allergy was misdiagnosed as infection. Multiple antibiotics failed to treat his erythema and pain. A cobalt allergy was diagnosed; however, prednisolone provided only mild relief. After 2 years, the patient chose bar removal over methotrexate treatment.

**Fig. 3:**
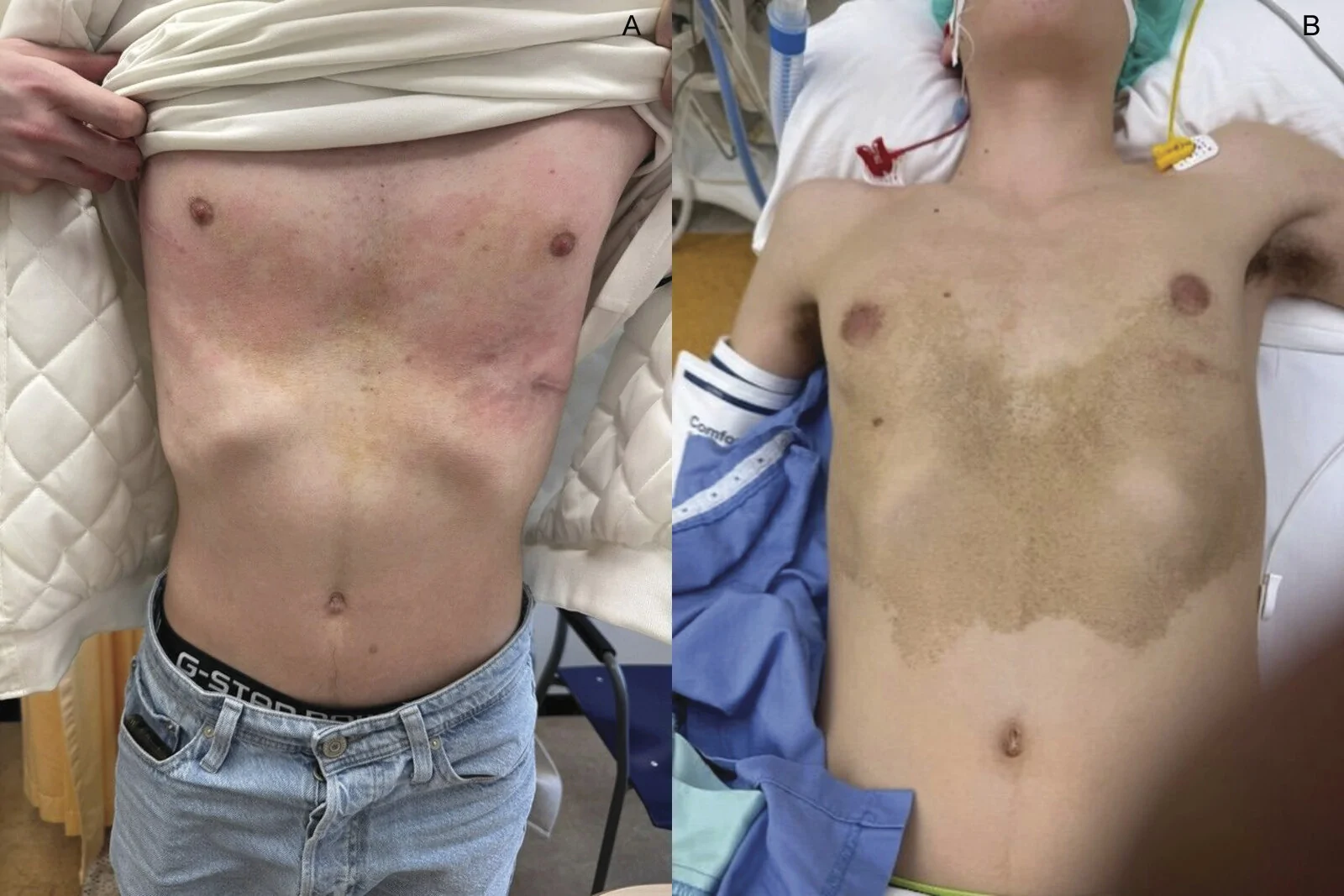
Terra firma-forme dermatosis. Early erythematous lesions ( **A** ). Late, brownish, dirt-like patches ( **B** ).

Terra firma-forme dermatosis, presenting as erythematous lesions that subsequently develop into brownish, dirt-like patches resistant to soap and water ( Fig. 3 ), occurred in 0.4% of patients ( *n* = 3/695) and was effectively treated with alcohol wipes.

## Discussion

Postoperative infections were found in 4.0% of the patients, with a shift from early- to late-onset infections over time. The most common infection symptoms were erythema and pain. CA became increasingly prevalent in the past decade. Infection occurred more often on the stabilizer plate side, and after bar dislocation and bar loosening. Bar removal operation times were longer with bar infections. Allergies can mimic infections. Terra firma-forme dermatosis is effectively treated with alcohol wipes.

### Incidence of Bar Infections

Our infection rate of 4.0% aligns with current literature (1.5–9.4%). 3 4 5 6 7 8 9 10 11 12 13 14 15 No consensus exists on acceptable infection rates in Nuss bar surgery. Even if the 1 to 5% threshold for clean wounds excluding prosthetic material is considered, our rate is still within this range, which is acceptable considering prosthetic operations have a higher threshold.

### Infection Management

Antibiotic regimens in our cohort were heterogeneous, limiting the ability to draw conclusions regarding treatment effectiveness, and reflecting evolving clinical practice and surgeon-dependent decision-making over the long study period. Similarly, studies describe a wide range of antibiotics, mostly based on the surgeon's preference, with SA being the most commonly cultured bacterium. 11 12 The latter study suggested oral clindamycin and cotrimoxazole for 1 to 2 months as long-term suppressive treatment. Rifampicin was also described in the literature. 4 Since SA is common, we recommend oral flucloxacillin plus biofilm-active rifampicin as first-line treatment for acute infections for 6 weeks. Cultures should be obtained before starting treatment. If a pathogen other than SA is isolated, antibiotics should be changed accordingly. Until bar removal, treat low-grade infections with oral amoxicillin or clindamycin. Shared decision-making and bar removal should be considered throughout this phase of treatment due to the risks of long-term antibiotic use. Use intravenous treatment only for clinically unstable individuals.

A schematic overview of our current institutional approach to infection management is provided in Supplementary Fig. S1 (available in the online version only). This overview is based on a combination of study findings and clinical experience but does not represent a standardized or validated treatment algorithm. Given the retrospective and heterogeneous nature of the data, it should be interpreted with caution and primarily serves as a basis for future prospective evaluation.

### Preoperative Measures

We recommend cefazolin preoperatively and for 24 hours postoperatively. 4 21 To minimize infection risk, we recommend following the international guidelines on prevention of surgical site infections. 18 19 The international guidelines for surgical site infections do not recommend adhesive drapes or double gloves, 20 and recent evidence on the effects of adhesive drapes, including antimicrobial ones, on prosthetic surgery infection rates is inconclusive. 22

### Symptoms, Imaging and Culturing

Erythema, pain, and exudate indicate infection, but fever is rare. Thoracic X-ray and, if inconclusive, a CT scan should be performed in all suspected cases to rule out bar dislocation, pneumonia, and pleural effusion. If these causes are excluded, ultrasound should be used to detect fistulas or fluid collections around the bar. When fluid is present, drainage and culture are essential for guiding antibiotics. Fistulas require surgical treatment.

In all suspected infections, cultures should be obtained before administering antibiotics, preferably fluid aspiration or intraoperative biopsies (at least five).

### Shift in Time of Infections and Type of Bacteria

SA was the most commonly cultured pathogen in early-onset Nuss bar infections. Late-onset CA infections, a slow-growing bacterium linked to indolent implant infections, have increased over the past decade. This may be due to improved surgical procedures lowering acute infections and increasing chronic, low-grade infections. Frequent and targeted testing may also increase CA detection. Since CA is a common cutaneous bacterium, it is commonly neglected unless implanted material is present. Surface swabs may detect CA even when it is not clinically relevant, leading to needless antibiotic therapy and delayed allergy diagnosis.

In our cohort, microbiological findings were interpreted in conjunction with clinical presentation and imaging evidence of implant involvement, rather than in isolation. However, due to the retrospective design, sampling techniques, incubation duration, and time to culture positivity were not standardized or consistently documented, limiting the ability to distinguish true infection from colonization. Future investigations should focus on culturing explanted bars to identify if CA is a pathogen or a contaminant in chronic infections.

### Factors Associated with Infections

Bar dislocation and stabilizer plate loosening were correlated to higher infection rates with 5.6 and 1.0% prevalence, respectively. Although these findings are based on univariate analyses and should be interpreted with caution, they support previous publications linking mechanical instability to postoperative infection risk. 23 Our findings suggest that mechanical instability may precede and contribute to the development of postoperative infections, rather than result from them. Infections were more frequent on the side with a stabilizer, which may reflect enhanced tissue manipulation or injury in these locations, potentially facilitating bacterial colonization. Such manipulation could alter the local tissue milieu, making latent or low-virulence bacteria like CA clinically relevant.

According to the literature, only the bridge technique has created a zero percent dislocation rate. 24 25 We mostly employ a single bar, making bridging impractical. Despite this, we have lowered our dislocation rate to 0 to 3.1% during the past 5 years. This improvement may be attributed to the expanding experience of our surgical team, 6 and the use of screw-fixed stabilizers and their medial placement, which reduces leverage and limits bar movement.

### Influence of Nuss Bar Infection on Bar Removal

Infections complicate the bar removal procedure, resulting in prolonged operative times. Bar removal and reimplantation after infection require multidisciplinary collaboration, particularly involving cardiac surgeons. The risks of infection and recurrent PE should be considered before reimplantation. A second surgery increases the risks due to scar tissue, which increases the risk of pericardial injury. Culture-based 2-week targeted antibiotic therapy with oral amoxicillin or clindamycin is recommended prior to bar replacement.

### Skin Reactions

If prolonged erythema without systemic infection markers, diagnostic imaging abnormalities, or unexplained postoperative inflammation occurs, allergies to implant materials (e.g., cobalt) should be investigated. A recent article describes delayed-onset postoperative grayish–brown hyperpigmentation, 26 which we believe is terra firma-forme dermatosis. A review noted it occurs more often near surgical sites, possibly triggered by surgery or cryoablation. 27 Though the cause is unclear, the pigmentation resists soap and water but is removed with 70% isopropyl alcohol. As these conditions were identified based on clinical suspicion rather than systematic screening, their reported incidence should be interpreted with caution.

## Future Directions and Limitations

The retrospective nature of our study introduces inherent biases and may lead to missing data due to nondigitized medical records. The findings of this study can be validated by using standardized follow-up protocols to improve techniques.

Due to the limited number of infection events ( *n* = 28), statistical power for subgroup analyses was limited, and multivariable analysis was not feasible without risk of overfitting. Associations between potential risk factors and infection should therefore be interpreted as descriptive rather than causal.

Additionally, the impact of sutures on infection development requires more research considering the use of hammocks. When sutures were utilized, early infections were more likely, suggesting they fostered bacterial colonization. Fixation methods should be optimized to reduce infection risk as surgical techniques advance.

## Conclusion

Postoperative infections had an incidence of 4.0%, with a shift from early- to late-onset infections over time. Bar dislocation and stabilizer plate loosening were associated with infection, emphasizing the need to optimize surgical procedures. Our proposed institutional infection management approach, based on these findings and clinical experience, emphasizes a structured diagnostic strategy, tailored antibiotic therapy, and multidisciplinary management, and will be prospectively evaluated. In cases with mild symptoms and negative cultures, or when CA is detected, alternative diagnoses such as implant allergy should be considered. Terra firma-forme dermatosis is easily treated with alcohol wipes.
