# Supplementary material for: Bar-Related Infections After Minimally Invasive Repair of Pectus Excavatum: A 25-Year Single-Center Retrospective Cohort Study
Source: Eur J Pediatr Surg. 2026 Jun 15;36(5):385–92. doi: 10.1055/a-2888-9665 (PMC13597110; doi:10.1055/a-2888-9665)
Supplement: Supplementary file 1 [file 10-1055-a-2888-9665_29629794.pdf]

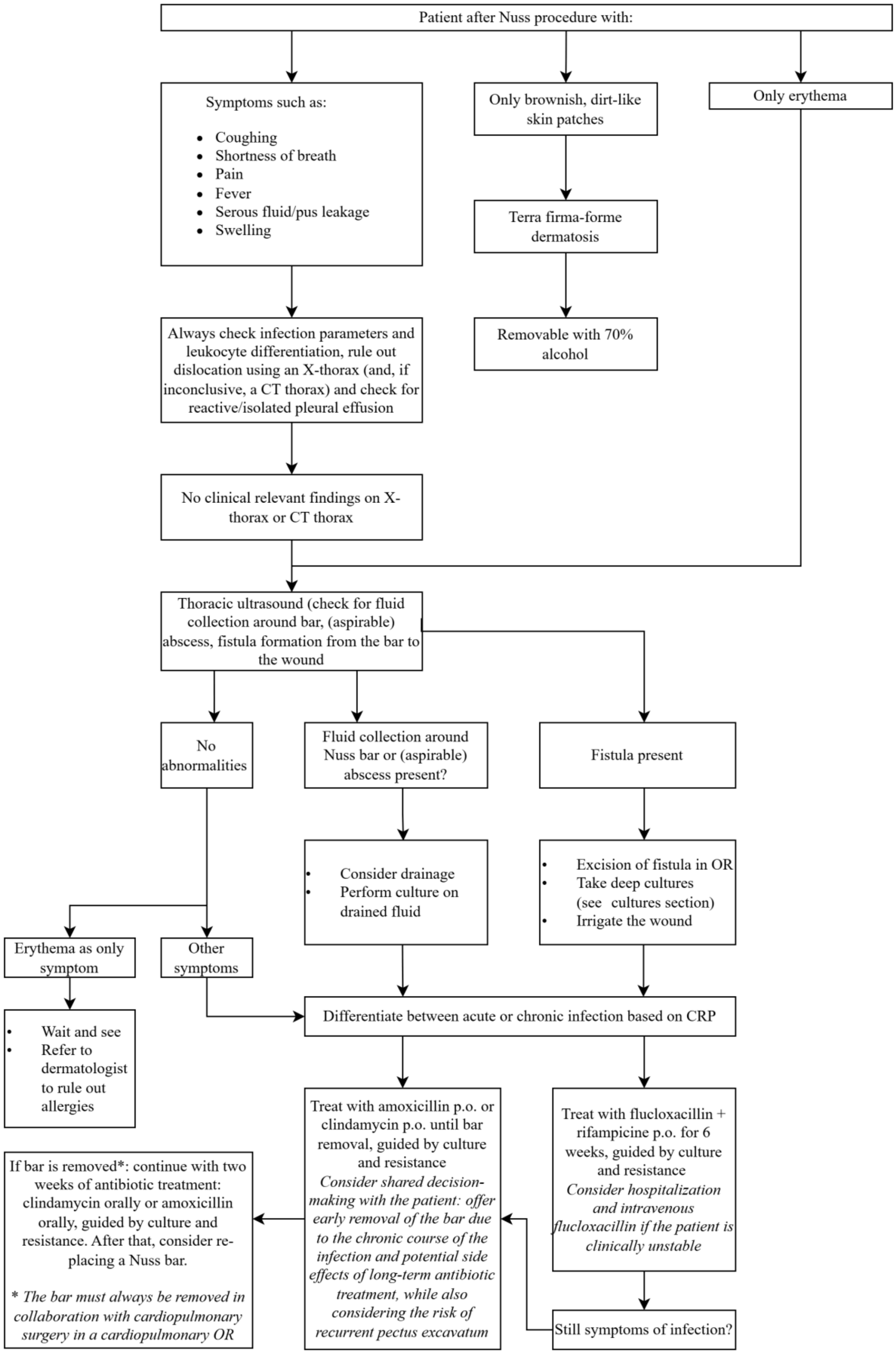
Patient after Nuss procedure with:
Symptoms such as:
• Coughing
• Shortness of breath
• Pain
• Fever
• Serous fluid/pus leakage
• Swelling
Only brownish, dirt-like skin patches
Only erythema
Terra firma-forme dermatosis
Removable with 70% alcohol
Always check infection parameters and leukocyte differentiation, rule out dislocation using an X-thorax (and, if inconclusive, a CT thorax) and check for reactive/isolated pleural effusion
No clinical relevant findings on X-thorax or CT thorax
Thoracic ultrasound (check for fluid collection around bar, (aspirable) abscess, fistula formation from the bar to the wound
No abnormalities
Fluid collection around Nuss bar or (aspirable) abscess present?
Fistula present
• Consider drainage
• Perform culture on drained fluid
• Excision of fistula in OR
• Take deep cultures (see cultures section)
• Irrigate the wound
Erythema as only symptom
Other symptoms
• Wait and see
• Refer to dermatologist to rule out allergies
Differentiate between acute or chronic infection based on CRP
Treat with amoxicillin p.o. or clindamycin p.o. until bar removal, guided by culture and resistance
Consider shared decision-making with the patient: offer early removal of the bar due to the chronic course of the infection and potential side effects of long-term antibiotic treatment, while also considering the risk of recurrent pectus excavatum
Treat with flucloxacillin + rifampicine p.o. for 6 weeks, guided by culture and resistance
Consider hospitalization and intravenous flucloxacillin if the patient is clinically unstable
Still symptoms of infection?
If bar is removed*: continue with two weeks of antibiotic treatment: clindamycin orally or amoxicillin orally, guided by culture and resistance. After that, consider re-placing a Nuss bar.
* *The bar must always be removed in collaboration with cardiopulmonary surgery in a cardiopulmonary OR*

Supplementary Fig. S1 Overview of institutional approach to suspected Nuss bar infection (CRP=C-reactive protein, OR=operation room).
